# Oxidative stress, telomere shortening, and DNA methylation in relation to low‐to‐moderate occupational exposure to welding fumes

**DOI:** 10.1002/em.21958

**Published:** 2015-05-27

**Authors:** Huiqi Li, Maria Hedmer, Tomasz Wojdacz, Mohammad Bakhtiar Hossain, Christian H. Lindh, Håkan Tinnerberg, Maria Albin, Karin Broberg

**Affiliations:** ^1^ Department of Laboratory Medicine, Section of Occupational and Environmental MedicineLund UniversityLundSweden; ^2^ Institute of Environmental Medicine, Karolinska InstitutetStockholmSweden

**Keywords:** particulate matter, welding fumes, oxidative stress, 8‐oxodG, telomere, DNA methylation

## Abstract

Evidence suggests that exposure to welding fumes is a risk factor for lung cancer. We examined relationships between low‐to‐moderate occupational exposure to particles from welding fumes and cancer‐related biomarkers for oxidative stress, changes in telomere length, and alterations in DNA methylation. We enrolled 101 welders and 127 controls (all currently nonsmoking men) from southern Sweden. We performed personal sampling of respirable dust and measured 8‐oxodG concentrations in urine using a simplified liquid chromatography tandem mass spectrometry method. Telomere length in peripheral blood was measured by quantitative polymerase chain reaction. Methylation status of 10 tumor suppressor genes was determined by methylation‐sensitive high‐resolution melting analysis. All analyses were adjusted for age, body mass index, previous smoking, passive smoking, current residence, and wood burning stove/boiler at home. Welders were exposed to respirable dust at 1.2 mg/m^3^ (standard deviation, 3.3 mg/m^3^; range, 0.1–19.3), whereas control exposures did not exceed 0.1 mg/m^3^ (*P* < 0.001). Welders and controls did not differ in 8‐oxodG levels (*β* = 1.2, *P* = 0.17) or relative telomere length (*β* = −0.053, *P* = 0.083) in adjusted models. Welders showed higher probability of adenomatous polyposis coli (*APC*) methylation in the unadjusted model (odds ratio = 14, *P* = 0.014), but this was not significant in the fully adjusted model (*P* = 0.052). Every working year as a welder was associated with 0.0066 units shorter telomeres (95% confidence interval −0.013 to −0.00053, *P* = 0.033). Although there were no clear associations between concentrations of respirable dust and the biomarkers, there were modest signs of associations between oxidative stress, telomere alterations, DNA methylation, and occupational exposure to low‐to‐moderate levels of particles. Environ. Mol. Mutagen. 56:684–693, 2015. © 2015 The Authors. Environmental and Molecular Mutagenesis Published by Wiley Periodicals, Inc.

## INTRODUCTION

Approximately 16,000 workers in Sweden are welders by occupation, and several million people worldwide are occupationally exposed to welding fumes [Antonini, 2003; IVL Svenska Miljöinstitutet]. Welding fumes have been categorized as a possible human carcinogen (Group 2B) [IARC, 1990], and studies show a 25%–40% increase in risk (measured as odds ratio) for lung cancer in welders [Hansen et al., 1996; Ambroise et al., 2006; 't Mannetje et al., 2012; Vallieres et al., 2012; Kendzia et al., 2013]. However, negative results have also been reported in large cohorts from Sweden and other countries [Sjögren et al., 1987; Danielsen et al., 2000; Gustavsson et al., 2000]. Welding fumes are derived from combustion, and contain a mixture of metal oxide particles; mild steel generates welding fumes mainly consisting of iron and manganese, but stainless steel generates fumes that also contain chromium and nickel [Leonard et al., 2010]. It has been suggested that the increased cancer risk is only related to welding with stainless steel [Simonato et al., 1991]. Nevertheless, some reports have shown that welding of mild steel is associated with increased cancer risk [Hansen et al., 1996; Ambroise et al., 2006].

Welding fumes consist of metal and metal oxide particles, with the particle size typically within the range of fine to ultrafine particles [Antonini, 2003; Hedmer et al., 2014]. Exposure to welding fumes can induce free radical activity in tissues [Leonard et al., 2010; Liu et al., 2013]. Increased oxidative stress in response to exposure to particles has been reported through the analysis of various biomarkers, including thiobarbituric acid reactive substances, protein carbonyls, protein sulfhydryls, glutathione peroxidase, manganese superoxide dismutase, and total antioxidant status [Fidan et al., 2005; Han et al., 2005]. In the human body, free radicals can cause DNA damage, which has been suggested as a possible mechanism for cancer development induced by exposure to welding fumes [Chuang et al., 2010; du Plessis et al., 2010]. One study showed that oxidative stress, measured as 8‐oxo‐7,8‐dihydro‐2′‐deoxyguanosine (8‐oxodG) in urine, predicted lung cancer risk among nonsmokers [Loft et al., 2006]. In addition, telomeres, the DNA–protein structures consisting of tandem repeats of TTAGGG at the end of eukaryotic chromosomes, play a key role in chromosome stability and may serve as biomarkers of cancer risk [Blasco, 2005]. For example, short telomeres in peripheral blood are associated with increased risk of various types of cancer, including lung cancer [Wu et al., 2003; Jang et al., 2008; Ma et al., 2011]. However, prospective studies have reported that long telomeres are associated with increased lung cancer risk [Shen et al., 2011; Lan et al., 2013; Seow et al., 2014].

In addition to causing genotoxic changes in the primary DNA sequence, particles might also affect chromatin modifications, which are referred to as epigenotoxic changes. For example, one key regulator of gene expression is 5‐methylcytosine DNA methylation. Hypermethylation of tumor suppressor genes (which turns off their gene expression) often occurs as an early change during carcinogenesis both in the tumor tissue and in body fluids, including peripheral blood [Esteller, 2008; Balgkouranidou et al., 2013]. Methylation of tumor suppressor genes can, therefore, be used as an epigenotoxic biomarker for early detection of cancer‐related changes [Ting et al., 2006; Iwamoto et al., 2011]. Aberrant DNA methylation patterns in patients with lung cancer have been reported in variant genes, including homeobox A9 (*HOXA9*) (previously analyzed in sputum specimens) [Hwang et al., 2011], cyclin‐dependent kinase inhibitor 2A (*CDKN2A*) (peripheral blood and tumor) [Vaissiere et al., 2009; Tan et al., 2013], short stature homeobox 2 (*SHOX2*) (plasma) [Kneip et al., 2011], adenomatous polyposis coli (*APC*) (sputum specimens) [Konno et al., 2004], O‐6‐methylguanine‐DNA methyltransferase (*MGMT*) (serum and sputum specimens) [Esteller et al., 1999; Guzman et al., 2012], cadherin 1, Type 1, E‐cadherin (*CDH1*) (sputum specimens) [Guzman et al., 2012], Dorsocross1 (*DOC1*), Ras association (RalGDS/AF‐6) domain family member 1A (*RASSF1A*) (peripheral blood and tumor tissue) [Vaissiere et al., 2009; Tan et al., 2013], homeobox B13 (*HOXB13*) (cell culture) [Rauch et al., 2006], and BCL2/adenovirus E1B 19kDa interacting protein 3 (*BNIP3*) (tumor) [Castro et al., 2010].

The aim of this study was to elucidate whether workers occupationally exposed to welding fumes show signs of oxidative stress measured in urine, telomere alterations in blood, and changes in DNA methylation of tumor suppressor genes in blood. We performed this study on welders and controls working in southern Sweden.

## MATERIALS AND METHODS

### Study Participants

We contacted 10 companies engaged in welding in southern Sweden. The 10 companies were medium‐sized and used welding mainly with mild steel to produce different machines and items (Table 1). All companies used the same common method, namely gas metal arc welding. We enrolled 101 welders, with the number of welders participating from each individual company ranging from 4 to 24. We recruited 127 controls from seven companies, of which six companies were storage houses organizing goods, and one company was a housing company working with gardening. The inclusion criteria for welders and controls were to be male and currently nonsmoking, and all participants had been nonsmoking for more than 1 year. Structured interviews were conducted by a trained nurse to obtain information about ethnicity (participants’ and their parents’ nationality), education (five categories), prescribed and nonprescribed medication, personal disease history (cancer, cardiovascular disease, respiratory disease, urinary disease, diabetes, and inflammation), family disease history (cancer and cardiovascular disease), diet (frequency of intake of vegetable and fruit with eight categories for each, and frequency of fish intake with seven categories), physical activity (four categories), previous smoking (yes or no, and if yes, starting and ending year of smoking), passive smoking (yes or no, and if yes, the source of passive smoking), use of snus (yes or no; snus is a moist powder tobacco product commonly consumed in Sweden), alcohol consumption (wine consumption and other alcohol consumption with six categories for each), current residence (size of town with four categories), wood burning stove/boiler at home (yes or no), wood smoke from the neighbourhood (yes or no), exposure to traffic (traffic intensity beside residence and time spent in traffic every day), work task, protection device, other exposures at work, occupational history, and hobbies related to particle exposure. All study participants answered the same questionnaire, with the exception of questions relating to work tasks, which differed between occupation groups.

**Table 1:** Personal Airborne Exposure to Respirable Dust for Welders^a^

			Mass concentration of respirable dust (mg/m^3^)		
Company	Manufactured product	No. of workers in the exposure measurements	GM	GSD	Range
1	Hydraulic lifting tables	5	1.2	2.6	0.5–3.6
2	Containers	6	0.9	2.8	0.2–4.3
3	Stoves	6	0.6	2.9	0.2–2.3
4	Heating boilers and pumps	10	0.3	2.9	0.1–2.2
5	Fork‐lift trucks	9	3.6	2.8	0.5–11.8
6	Dumper‐trucks	9	1.9	2.0	0.5–5.8
7	Wheel loaders	9	1.7	2.7	0.5–9.1
8	Equipment for mining industry	4	1.0	2.0	0.5–2.3
9	Railway wagons	7	2.2	2.9	0.8–19.3
10	Asphalt rollers	5	1.2	2.3	0.6–3.3
Total		70	1.2	3.3	0.1–19.3

a The Swedish occupational exposure limit for respirable dust is 5 mg/m^3^.

GM, geometric mean; GSD, geometric standard deviation.

Venous blood and spot urine samples were collected from each participant. Blood was collected at the time of the interview, and urine was collected during the last 4 hrs of an 8‐hr work shift throughout the week. All study participants gave their informed written consent to take part in the study, and the study was approved by the Regional Ethical Committee of Lund University, Sweden.

### Exposure Assessment

For practical reasons, it was not possible to perform exposure measurements on all 101 welders. Fifty‐three welders underwent exposure measurements, and the levels of exposure for the remaining 48 welders were estimated based on exposure data from the measured welders. Seventeen welders who were not included in the interview and biological sampling also took part in the exposure measurement. The exposure data from these 17 welders were used to obtain more accurate exposure assessment.

#### Measurement of Respirable Dust

Measurements of respirable dust were performed once in each of the welding companies on a different day from the interview and biological sampling. Samples were collected from within the workers’ breathing zones. Approximately half of the welders were using powered air purifying respirators (PAPRs). For these welders, the air outside the PAPRs was sampled. Respirable dust was collected using cyclones (BGI4L, BGI, Waltham, MA; 50% cut‐off at an aerodynamic equivalent particle diameter of 4 μm) on preweighed 37 mm mixed cellulose ester filters (0.8 µm pore size) in cassettes (Sure‐Seal, Omegatech, Pittsburg, PA). Sampling pumps (MSA Escort Elf, Pittsburgh, PA) were operated at a flow rate of 2.2 L/min. The airflow was regularly checked with a primary calibrator (TSI Model 4199, TSI, Shoreview, MN) before, during, and after the sampling. The average sampling time was 6.8 hr (range, 2.4–8.6 hr). The filter samples were analyzed gravimetrically according to a certified method with a limit of detection of 0.05 mg/sample.

To assess the workplace protection factor for the PAPRs, we performed parallel measurements of respirable dust inside and outside the protection on three workers from different companies. The set‐up has recently been published elsewhere [Hedmer et al., 2014]. The parallel samplings showed that the respirable dust concentrations were at least three times lower inside the PAPRs. Based on this result and literature data on workplace protection factors [Goller and Paik, 1985; Han, 2002; Janssen et al., 2007; Hedmer et al., 2014], the concentrations of respirable dust measured outside the PAPRs were then reduced by a factor 3 to estimate exposure inside the PAPRs.

Nineteen workers at two control companies were monitored for respirable dust. The average sampling time for the controls was 7.2 hr. Stationary measurements of respirable dust were conducted at another four control companies with a direct‐reading instrument (Sidepak Model AM510, TSI).

#### Assessment of Exposure to Respirable Dust

The assessment was done individually for each of the 48 welders by reference to data from welders working at the same location, engaged in similar tasks, and in the same company. Moreover, exposure data previously collected at the companies were also used in the exposure assessments [Hedmer et al., 2014]. There were two participants who could not be assessed: one welder reported that he worked 100% with soldering/brazing, which seemed unreasonable; another welder's information about the local exhaust ventilation was missing; therefore, it was difficult to correctly estimate his exposure. These two participants were treated as missing for respirable dust.

### 8‐OxodG Measurement

Urinary 8‐oxodG is produced by an interaction between hydroxyl radicals and 2′‐deoxyguanosine. This lesion is usually removed from the body and excreted in urine, and 8‐oxodG is considered as a marker of oxidative stress [Cooke et al., 2009]. Concentrations of urinary 8‐oxodG were measured with a simplified method by liquid chromatography tandem mass spectrometry. Internal standard (15N5‐8‐oxodG, 5 pmol) was added directly to 100 µL of urine, and then ultrapure water was added to make a final volume of 1 mL, prior to quantitative analysis by a triple quadruple linear iron trap mass spectrophotometer (QTRAP 5500; AB SCIEX, Applied Biosystems, Foster City, CA) equipped with a Turbo Ion‐Spray source coupled to a high‐speed liquid chromatography system with four pumps (Prominence UFLC, UFLCXR; Shimadzu Corporation, Kyoto, Japan). MS analyses were carried out using selected reaction monitoring in the positive ion mode. 8‐oxodG concentrations were determined by peak area ratios between 8‐oxodG and the internal standard 15N5‐8‐oxodG. Urine samples were processed and analyzed in duplicate on two occasions; the repeatability of the method, expressed as coefficient of variance (CV), was 10%. Three internal controls (urine samples from Swedish adults) were included in each batch for which the CVs were 12%, 9%, and 4%, respectively. The 8‐oxodG concentrations were above the limit of detection (0.5 nmol/L) in all urine samples. The concentration of urinary 8‐oxodG was adjusted to urinary specific gravity.

### Telomere Length

DNA was isolated from whole peripheral blood by the Qiagen DNA Blood Midi kit (Qiagen, Heidelberg, Germany). Quantitative polymerase chain reaction (PCR) was used to determine relative telomere length, as described previously [Li et al., 2012]. Briefly, master mixes for telomere runs were prepared with telomere primers (0.45 μM of each primer), 1 × PCR Buffer (Life Technologies, Carlsbad, CA), 1.75 mM MgCl_2_, 0.8 mM dNTPs, 0.3 mM SybrGreen (Life Technologies), 1 × Rox (Life Technologies), and 0.5 U *Taq*Platina (Life Technologies). Master mix for haemoglobin beta chain (*HBB*) gene runs were prepared with *HBB* primers (0.40 μM for each primer) and KAPA SYBR FAST qPCR Kit Master Mix (2 ×) ABI Prism (Kapa Biosystems, Woburn, MA). The PCR was performed on a real‐time PCR machine (7900HT, Applied Biosystems). Five microliters of sample DNA (4 ng/μL) was added to each reaction mixture (final volume 20 μL). A standard curve, a reference DNA, and a negative control were also included in each run, all run in triplicate. For the standard curve, one calibrator DNA sample was diluted serially by twofold per dilution, to produce concentrations of 1–16 ng/μL. *R* ^2^ for each standard curve was >0.99. Standard deviations (for *C_t_* values) were accepted at <0.1. The relative length of the telomeres was obtained through calculating the ratio (*T*/*S*) of telomere repeats product and single copy gene product (*S*, here *HBB*) for each individual by the formula *T*/*S* = 2^−Δ^ *^Ct^*, where Δ*C_t_* = *C_t_*_ telomere_ − *C_t _*_HBB_. This ratio was then compared with the ratio of the reference DNA. The telomere length ratio is an arbitrary value. The CV of 12 runs was 7%.

### DNA Methylation

Bisulfite modification was performed on 500 ng/sample of the template DNA with EZ‐96 DNA Methylation‐Gold kit (Zymoresearch, Irvine, CA) according to the manufacturer's protocol. Controls included methylated (Universal Methylated Human DNA Standard, Zymoresearch) and nonmethylated (EpiTect Control DNA, Qiagen) DNA, and they were prepared following the same procedure as the DNA samples. Methylation‐sensitive high‐resolution melting (MS‐HRM) assays were designed according to the guidelines described earlier [Wojdacz et al., 2008]. Each PCR reaction was performed in triplicate with PCR mix containing 1 × MeltDoctor HRM Master Mix (Life Technologies), 500 nM of each primer, and 15 ng of the template (theoretical calculation after bisulfite modification). All MS‐HRM results were aligned according to the melting curve of the controls. The controls consisted of mixtures of the reference samples: 0.1%, 1%, and 10% of methylated template in a nonmethylated background, and 100% fully methylated template. PCR amplification was performed on a real‐time PCR machine (7900HT, Applied Biosystems) with the parameters for amplification and HRM analyses: hot start for 10 min at 95°C; 50 cycles of 95°C for 15 sec; annealing at optimal primer annealing temperature for 10 sec; and elongation at 70°C for 20 sec. The HRM analysis consisted of denaturation at 95°C for 15 sec at ramp rate 100; re‐annealing at 60°C for 1 min at ramp rate 100; and melting from 60°C to 95°C at ramp rate 1. The MS‐HRM assays were calibrated to detect methylated alleles at the level of 15 pg in the background of nonmethylated alleles. HRM in this study was used as a qualitative method, to assay presence or absence of methylation in each sample, irrespective of level of methylation. Samples were thereby categorized as methylated (cut‐off was > 0% methylation) or nonmethylated, because the levels of methylation, in general, were low in this study. It was confirmed by sequencing (*N* = 8) that samples showing any methylation according to this criterion were methylated, and that samples that did not show methylation were not methylated. The average level of methylation for methylated samples was around 1%. HRM data were analyzed using the SDS 2.4.1 software (Life Technologies).

### Analyses of Inflammatory Response

C‐reactive protein and serum amyloid A were analyzed at the Department of Clinical Chemistry in Lund University Hospital by the use of immunoturbidimetry and immunonephelometry, respectively, via standard protocols.

### Statistical Analyses

DNA methylation was categorized as low if one gene was methylated in the participant, medium if two genes were methylated, or high if three or more genes were methylated (methylation index). Medians and 5–95 percentiles of age, body mass index (BMI), prevalence of characteristics, and levels of biomarkers were calculated. The differences among groups were compared by t‐test for continuous variables or Fisher's exact test for categorical variables.

General linear model was used to identify associations between 8‐oxodG and telomere length versus exposure. Ordinal regression was used for associations between methylation index versus exposure. Logistic regression was used for associations between DNA methylation versus exposure. The associations between the effects and working years as welder or concentration of respirable dust were analyzed in the welders only. Possible confounders were considered and included as adjustments, such as age, BMI, previous smoking, passive smoking, current residence, and wood burning stove/boiler at home.

All statistical analyses were conducted using SPSS 21.0 (SPSS, Chicago, IL), and statistical significance was defined as *P* < 0.05 (two‐tailed).

## RESULTS

### Occupational Exposure

The levels of exposure are listed in Table 1. Welders had been working at their current companies for an average of 7.0 years (range, 0–45 years), and only nine welders had been working for less than 12 months. Most welders worked with mild steel; 11 welders worked with stainless steel, of whom eight worked both with mild and stainless steel, and three worked with stainless steel only. The welders were exposed to welding fumes, with an average concentration of 1.2 mg/m^3^ measured as respirable dust, with a range of 0.1–19.3 mg/m^3^. Out of 101 welders, 97 had exposure lower than the Swedish occupational exposure limit for respirable dust (5 mg/m^3^) [Swedish Work Environment Authority, 2011]. The controls had no occupational contact with welding fumes. The results of personal exposure measurements showed that no participants in the control group were exposed to respirable dust above 0.1 mg/m^3^. Stationary measurements of respirable dust showed similar results to personal samplings (lower than 0.1 mg/m^3^). The difference in respirable dust exposure between welders and controls was significant (*P* < 0.001).

### General Characteristics of the Study Participants

Most individual characteristics were similar when comparing welders with controls (Table 2). The majority (96%) reported having European ancestry, and there was no difference in ethnicity between the groups (*P* = 0.75). Most participants had their highest achieved level of education as high school or lower (89%). There was also no difference between groups in their own or family history of cancer, prescribed medication, vegetable, fruit or fish intake, use of snus, wine consumption, or daily time spent in traffic. All participants were current nonsmokers, and the percentages of previous smoking between groups were similar (*P* = 0.22). However, the pattern of exposure to passive smoking was significantly different (*P* = 0.0041), with more welders exposed to passive smoking. Use of a wood burning stove/boiler at home was also more common in welders (*P* < 0.001). Finally, a larger proportion of controls lived in cities rather than in small towns (*P* = 0.010).

**Table 2:** Basic Characteristics of the Male Workers, Including Biomarkers of Genotoxicity and Epigenotoxicity

	Controls	Welders	*P*‐value
Age^a^	43 (23–56)	41 (23–60)	0.93^b^
BMI^a^	27 (22–34)	28 (22–34)	0.70^b^
Ethnicity (parents’ nationality in European/others)	122/5 (3.9%)	96/5 (5.0%)	0.75^c^
Education (high school or lower/university or higher)	109/17 (87%)	94/7 (93%)	0.13^c^
Years working in current occupation^a^	6.0 (0.83–24)	7.0 (0.50–24)	0.50^b^
History of cancer (yes/no)	2/125	0/101	0.50^c^
Family history of cancer (yes/no/do not know)	26/95/5	16/80/5	0.66^c^
Previous smoking (yes/no)	43/83 (34%)	43/58 (43%)	0.22^c^
Passive smoking (yes/no)	16/111 (13%)	29/72 (29%)	0.0041^c^
Current residence (big city/small city/big town/small town)	29/28/52/18	10/16/49/26	0.010
Wood burning stove/boiler at home (yes/no)	27/100 (21%)	47/54 (47%)	<0.001^c^
8‐OxodG^a^(nmol/L)	13 (6.2–20)	14 (7.0–28)	0.070^b^
Relative telomere length^a^	0.88 (0.57–1.3)	0.86 (0.55–1.2)	0.090^b^
DNA methylation index^d^ (low/medium/high)	39/51/30	26/34/28	0.59^c^
*HOXA9* methylation^e^	116/10 (92%)	95/2 (98%)	0.072^c^
*SHOX2* methylation^e^	59/63 (48%)	42/56 (43%)	0.50^c^
*CDKN2A* methylation^e^	46/80 (37%)	43/58 (43%)	0.41^c^
*MGMT* methylation^e^	20/107 (16%)	16/85 (16%)	1.0^c^
*APC* methylation^e^	1/126 (0.79%)	9/84 (9.7%)	0.0022^c^
C‐reactive protein^a^ (mg/L)	1.1 (0.30–5.0)	1.2 (0.30–5.0)	0.60
Serum amyloid A^a^ (mg/L)	2.4 (0.40–14)	2.3 (0.95–8.7)	0.60

a Median (5%–95%).

b The *P*‐values were from t‐test.

c The *P*‐values were from Fisher's exact test.

d Number of genes that were methylated (low: one gene; medium: two genes; and high: three or more genes).

e Methylated/nonmethylated.

### Biomarkers of Genotoxicity and Epigenotoxicity

Data on levels of 8‐oxodG and telomere length were collected from all participants. The mean concentration of urinary 8‐oxodG was 14 nmol/L (standard deviation, 5.8), and the mean relative telomere length was 0.89 (standard deviation, 0.21). Examination of *BNIP3* failed in our analysis; three genes (*DOC1, RASSF1A*, and *HOXB13*) were nonmethylated in all participants, and *CDH1* was methylated only in one participant; therefore, these five genes were excluded from further analysis. The prevalence of methylation in blood of the remaining five genes varied between different genes from 4.3% to 95% (Table 2). Levels of 8‐oxodG and relative telomere lengths were not different between welders and controls (*P* = 0.070 and 0.090, Table 2). However, DNA methylation in blood differed for the gene *APC* (*P* = 0.0022), with significantly more welders showing methylation compared with controls. The correlations between 8‐oxodG, telomere length, and DNA methylation versus characteristics, as well as the correlations among the biomarkers were explored by Spearman's correlation (Supporting Information Table S1 and Table S2).

General linear model was adopted to analyze differences in biomarkers of genotoxicity and epigenotoxicity between exposure groups, with and without adjustments (Table 3). The differences in 8‐oxodG and telomere length between groups were not significant. The frequency of *APC* methylation was significantly different between welders and controls in the unadjusted model, as the welders showed a higher proportion of methylation. When adjustments (age, BMI, previous smoking, passive smoking, current residence, and wood burning stove/boiler at home) were included, the odds ratio of *APC* methylation was decreased by 36% (*P* = 0.052), and this attenuation was mainly due to adding current residence and wood burning at home to the model. We also included biomarkers of infection and inflammation (C‐reactive protein and serum amyloid A in blood) in the model as a sensitivity analysis, but they did not change the effect estimates or *P*‐values (data not shown).

**Table 3:** Difference in Biomarkers of Genotoxicity and Epigenotoxicity Between Welders and Controls

		Unadjusted			Adjusted^a^		
		Effect	95% CI	*P*	Effect	95% CI	*P*
8‐oxodG^b^	Occupation groups	1.4	−0.12–2.9	0.070	1.2	−0.48–2.8	0.17
Telomere length^b^	Occupation groups	−0.048	−0.10–0.0075	0.090	−0.053	−0.11–0.0071	0.083
DNA methylation index^c^	Occupation groups	1.3	0.76–2.1	0.37	1.2	0.69–2.09	0.52
*HOXA9* methylation^d^	Occupation groups	4.1	0.88–19	0.073	3.7	0.73–19	0.11
*SHOX2* methylation^d^	Occupation groups	0.80	0.47–1.4	0.42	0.84	0.46–1.5	0.56
*CDKN2A* methylation^d^	Occupation groups	1.3	0.75–2.2	0.35	1.1	0.62–2.0	0.71
*MGMT* methylation^d^	Occupation groups	1.0	0.49–2.1	0.99	1.3	0.58–2.7	0.57
*APC* methylation^d^	Occupation groups	14	1.7–109	0.014	8.9	0.98–81	0.052

a Adjustment included age, BMI, previous smoking, passive smoking, current residence, and wood burning stove/boiler at home.

b General linear model with effect as beta estimate.

c Ordinal regression model with effect as odds ratio.

d Logistic regression model with effect as odds ratio.

**Figure 1:**
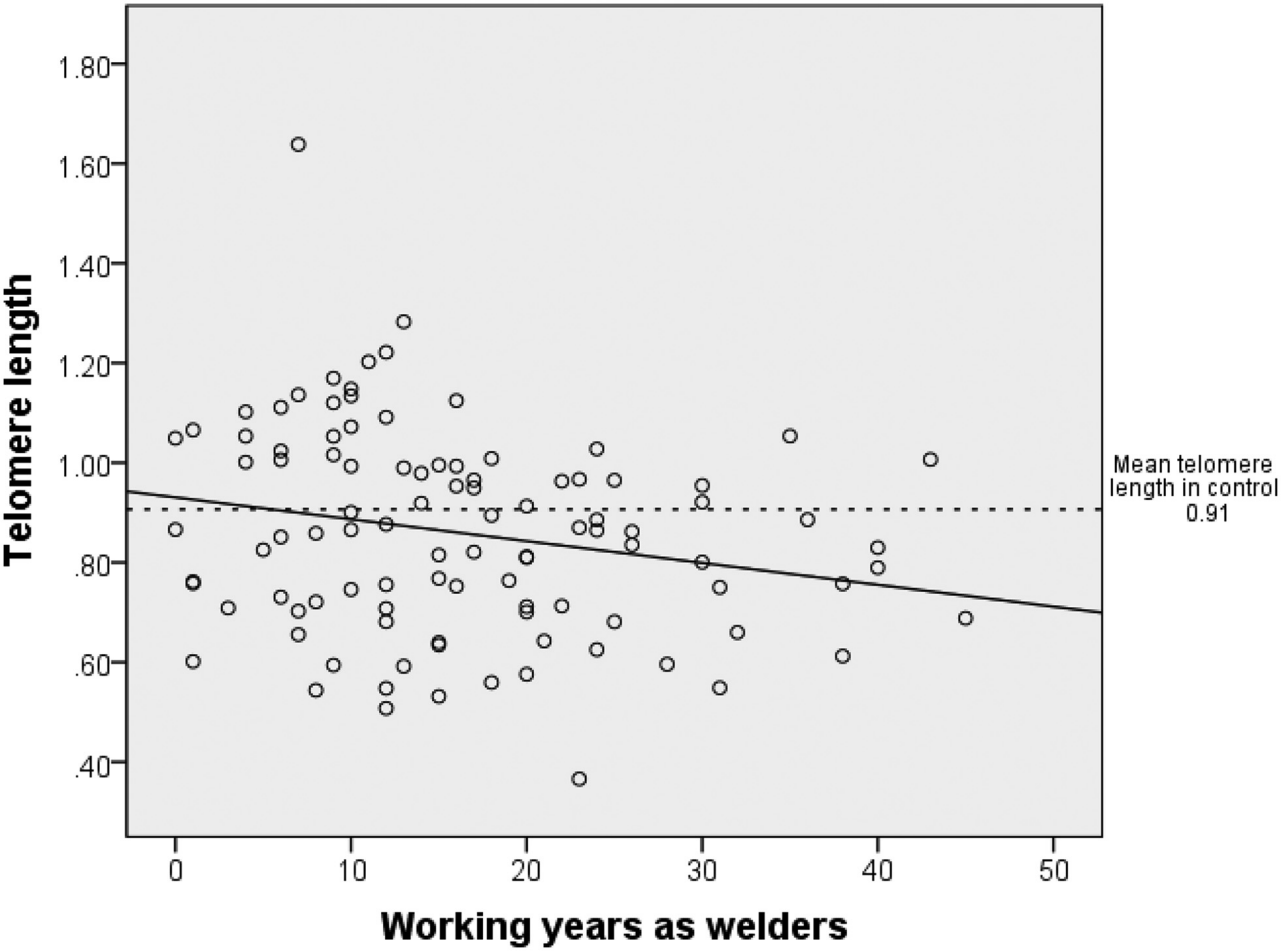
Scatterplot of working years as a welder and telomere length in welders. Solid line represents the regression fit between working years as a welder and telomere length. Dotted line represents the mean telomere length in the control group.

Welders were analyzed for associations between biomarkers of genotoxicity and epigenotoxicity, and the number of working years as welder (Table 4 and Fig. 1). More working years as a welder was associated with shorter telomeres (*P* = 0.026) in the unadjusted model. Age was not associated with telomere length in welders (*β* = −0.0015, 95% confidence interval, −0.0051 to 0.0022, *P* = 0.43). After adjusting for age (working years as a welder was correlated with age: Pearson's correlation = 0.75, *P* < 0.001) and other possible confounders, the association between telomere length and working years as a welder was still significant, and the size of the effect was also similar (*P* = 0.033).

**Table 4:** Associations Between Biomarkers of Genotoxicity and Epigenotoxicity and Working Years as a Welder

		Unadjusted			Adjusted^a^		
		Effect	95% CI	*P*	Effect	95% CI	*P*
8‐oxodG^a^	Working years	0.0080	−0.11 to 0.13	0.89	0.092	−0.099 to 0.28	0.34
Telomere length^a^	Working years	−0.0044	−0.0082 to −0.00053	0.026	−0.0066	−0.013 to −0.00053	0.033
DNA methylation index^b^	Working years	1.0	0.99 to 1.07	0.21	1.0	0.95 to 1.1	0.77
*HOXA9* methylation^c^	Working years	1.0	0.88 to 1.2	0.86	0.63	0.29 to 1.4	0.25
*SHOX2* methylation^c^	Working years	1.0	0.98 to 1.1	0.34	0.99	0.93 to 1.1	0.76
*CDKN2A* methylation^c^	Working years	0.98	0.94 to 1.0	0.28	0.99	0.93 to 1.1	0.82
*MGMT* methylation^c^	Working years	1.1	1.0 to 1.1	0.0040	1.0	0.95 to 1.1	0.36
*APC* methylation^c^	Working years	1.0	0.97 to 1.1	0.25	1.1	0.93 to 1.2	0.41

Adjustment included age, BMI, previous smoking, passive smoking, current residence, and wood burning stove/boiler at home.

a General linear model with effect as beta estimate.

b Ordinal regression model with effect as odds ratio.

c Logistic regression model with effect as odds ratio.

Associations between biomarkers and concentrations of respirable dust (based on exposure assessment) were also investigated in welders (Table 5). No significant associations were found for the biomarkers, without or with adjustments.

**Table 5:** Associations Between Biomarkers of Genotoxicity and Epigenotoxicity and Concentration of Respirable Dust in Welders

		Unadjusted			Adjusted^a^		
		Effect	95% CI	*P*	Effect	95% CI	*P*
8‐oxodG^b^	Respirable dust	0.23	−0.53 to 0.99	0.55	0.25	−0.55 to 1.1	0.53
Telomere length^b^	Respirable dust	−0.0026	−0.028 to 0.023	0.84	0.0027	−0.023 to 0.028	0.84
DNA methylation index^c^	Respirable dust	1.0	0.80 to 1.26	0.99	1.0	0.78 to 1.3	0.93
*HOXA9* methylation^d^	Respirable dust	6.8	0.054 to 861	0.44	7.2	0.030 to 1,745	0.48
*SHOX2* methylation^d^	Respirable dust	1.0	0.79 to 1.3	0.91	1.0	0.77 to 1.3	0.95
*CDKN2A* methylation^d^	Respirable dust	1.1	0.87 to 1.4	0.42	1.1	0.87 to 1.5	0.34
*MGMT* methylation^d^	Respirable dust	0.90	0.61 to 1.3	0.60	0.77	0.52 to 1.1	0.17
*APC* methylation^d^	Respirable dust	0.35	0.078 to 1.6	0.35	0.25	0.050 to 1.3	0.10

a Adjustment included age, BMI, previous smoking, passive smoking, current residence, and wood burning stove/boiler at home.

b General linear model with effect as beta estimate.

c Ordinal regression model with effect as odds ratio.

d Logistic regression model with effect as odds ratio.

## DISCUSSION

We found evidence of modest genotoxicity and epigenotoxicity in relation to occupational exposure to welding fumes. The strongest association was that welders who had worked for more years had shorter telomeres. Most other changes were subtle. Nevertheless, given that the exposure levels of workers in this study were, in general, below the Swedish occupational exposure limit, our results highlight that low‐to‐moderate levels of exposure to particles (which is very common in today's workplace) might increase workers’ risk for DNA damage and in turn cancer lesions.

This study had multiple advantages. Only current nonsmokers were recruited, as smoking is a known confounder for associations between exposure to fine particles and both genotoxicity and epigenotoxicity. Exposure to particles (including particles derived from nonworking exposure sources) was carefully evaluated from questionnaire data, as well as from the direct measurement of particle exposure in the different companies involved, and in controls. DNA methylation was measured by MS‐HRM, a sensitive and specific method for detection of methylation [Wojdacz and Dobrovic, 2007]. The 8‐oxodG and telomere length analysis showed low levels of methodological variation.

Our study also had some weaknesses. For example, cross‐sectional studies are generally vulnerable to confounders. Therefore, extensive information about potential confounders was collected from the participants. Nevertheless, it is still possible (though not very likely) that residual confounders did remain. In addition, exposure measurements could not be done on all study participants, and measurements were not performed on the same day as the interviews and biological samplings. The median time between the biological samplings and the exposure measurements was 2 months (range, 0–9 months). A further weakness of this study is that we analyzed telomere length and DNA methylation in peripheral blood as a proxy for how welding fume particles can damage lung tissue. However, peripheral blood is also considered a relevant target for welding fumes, as inflammation in the lungs induced by particle exposure may release small peptides or other substances into blood [Dubowsky et al., 2006], and particles themselves can also translocate into the blood stream and cause damage [Wallenborn et al., 2007]. Exposure‐related changes in DNA methylation or telomere length in peripheral blood could reflect alterations within cells, or could be due to an alteration of blood cell composition as a result of infection or inflammation [Steenhof et al., 2014]. However, we accounted for this by adjusting for concentrations of acute phase response markers in the blood as a proxy for altered blood cell composition due to infection or inflammation, and this sensitivity analysis did not change the results. Finally, 8‐oxodG was measured in urine. Although the organ and cellular sources for this lesion are not well known, the levels are considered to reflect the whole body production of 8‐oxodG.

Several markers of genotoxicity and epigenotoxicity were considered in this study, which might have resulted in false positives as a result of multiple comparisons. Telomere length changes and elevated 8‐oxodG have previously been reported in the literature in relation to particles and cancer risk [Blasco, 2005; Loft et al., 2006; Moller and Loft, 2010; Zhang et al., 2013], and were, therefore, considered as the main outcomes in this study, whereas analysis of particle‐induced DNA methylation was exploratory.

Although the different companies made different products, they generally used the same technique for welding. Thus, the compositions of welding fumes were rather homogenous [Hedmer et al., 2014]. At the time when the exposure measurements were performed, no minimized filter cassettes were available to be used within the PAPRs. A correction factor of 3 was applied to account for the use of PAPRs, because the respirable dust concentrations inside the PAPRs were at least three times lower than outside. The uncertainty in true exposure to respirable dust when wearing PAPRs could have resulted in an over‐estimation of the exposure. Finally, although there was a delay between biological sampling and exposure measurements, all of the workers that we analyzed had worked at the same task throughout the period.

Urinary 8‐oxodG is considered to be a reliable biomarker for oxidative stress induced by particle exposure; it has been studied in prospective studies, and one study found it to be predictive for lung cancer [Moller and Loft, 2010]. Higher oxidative stress and lower antioxidant capacity have been reported in welders [Stepnewski et al., 2003; Han et al., 2005; Liu et al., 2013], but results regarding urinary 8‐oxodG and exposure to welding fumes were not consistent. Nuernberg et al. [2008] reported a significant increase in urinary 8‐oxodG after 6 hrs of exposure to welding fumes, with an average concentration of 0.82 mg/m^3^, which is lower than that in our study. In contrast, Liu et al. [2013] did not find any significant difference in 8‐oxodG between welders and controls (though exposure levels were not reported in that study). Although 8‐oxodG has a short turn‐over in the body [Nuernberg et al., 2008], the urine samples for our study were collected during the last 4 hrs of an 8‐hr work shift, and should thus reflect changes induced by short‐term exposures. Therefore, the effects of welding particles on 8‐oxodG appear to be rather marginal. To further evaluate cancer‐related effects from occupational particle exposure, we, therefore, evaluated biomarkers that better reflect long‐term effects: telomere length and changes in DNA methylation.

Telomere length in blood was marginally shorter in welders than in controls and significantly shorter in relation to working years as a welder. Studies on telomere length and particulate matter [Hoxha et al., 2009; McCracken et al., 2010; Dioni et al., 2011; Hou et al., 2012] suggest that short‐term exposure induces longer telomeres and long‐term exposure induces shorter telomeres. In our study, most of the welders had been working as welders for more than 1 year and, thus, can be regarded as having long‐term exposure. The finding of shorter telomeres in welders suggests that this exposure might be carcinogenic, as short telomeres in peripheral blood have been associated with various types of cancer, including lung cancer [Ma et al., 2011; Willeit et al., 2011]. In contrast, other studies have reported that longer telomeres are also associated with increased lung cancer risk [Shen et al., 2011; Lan et al., 2013; Seow et al., 2014]. One can, therefore, assume that either long or short telomeres may elevate cancer risks depending on the specific cellular history of somatic mutations. In cells where the pathways for senescence and apoptosis are blocked (e.g., by mutations in key genes), cells with short telomeres would be prone to chromosomal instability, a key factor in carcinogenesis. Cells with longer telomeres can afford more rounds of cell division, leading to a higher probability of accumulating more somatic mutations, which in turn could inhibit senescence and apoptosis, and initiate carcinogenesis. Evidently, longitudinal studies of welders are warranted to clarify what the welding exposure‐related changes in telomere length means for future cancer risk.

We observed increased *APC* methylation in welders, consistent with a previous study that showed an increase in *APC* methylation in workers in an electric steel plant after 3 days of occupational exposure to metal‐rich particles with an average concentration of PM1 at 8.48 μg/m^3^ [Hou et al., 2011]. The tumor suppressor *APC* gene regulates the Wnt signaling pathway, which is very important in the control of cell growth [Sparks et al., 1998]. Hypermethylation of the *APC* promoter has been found in the serum and plasma of lung cancer patients, and in patients with other types of epithelial cancer, compared with healthy controls [Usadel et al., 2002]. Further, it should be noted that wood burning at home in our study was associated with increased methylation of *APC*, showing that different particles from combustion can stimulate *APC* methylation. The mechanism of particle exposure‐induced DNA methylation is not clearly understood, but it has been suggested that several pathways could be involved. Oxidative stress may be one possible mechanism for particulate matter‐induced hypermethylation of *CDKN2A*, resulting from the induction of the mitochondrial ROS‐JNK‐*DNMT1* pathway in mice [Soberanes et al., 2012]. Other mechanisms may include disturbance of the one‐carbon metabolism that produces methyl groups for DNA methylation, as it has been shown that exposure to particulate matter could lead to elevated levels of plasma homocysteine, a key metabolite in one‐carbon metabolism [Park et al., 2008].

We did not find any associations between genotoxicity, or epigenotoxicity, and concentration of respirable dust, suggesting that the effects on the biomarkers that we measured in this study reflect long‐term alterations. We used working years as a welder as a marker for long‐term exposure because we could not obtain detailed information on cumulative exposure. We believe that the average concentration of welding fumes within the workshop is stable over time, based on data from this study and previous studies [Hedmer et al., 2014]. However, as the exposure measurement was not done on the same day as the interview and sampling, this may limit the analysis of association between exposure and biomarkers.

## AUTHOR CONTRIBUTIONS

HL recruited all participants, measured telomere length, performed statistical analyses, and drafted the manuscript. MH carried out the exposure measurement and assessment. TW performed measurement of DNA methylation. MBH measured urinary 8‐oxodG. CHL supervised the method of 8‐oxodG measurement. HT supervised the exposure measurement and assessment. HT and MA helped with the study design and with locating appropriate study participants. KB designed this project and supervised the study. All authors revised the manuscript critically. All authors read and approved the final manuscript.

*Accepted by—*U. Vogel

## Supporting information

Supporting Information
