# Supplementary material for: Oxidative stress, telomere shortening, and DNA methylation in relation to low‐to‐moderate occupational exposure to welding fumes
Source: Environ Mol Mutagen. 2015 May 27;56(8):684–93. doi: 10.1002/em.21958 (PMC4755249; doi:10.1002/em.21958)
Supplement: Supplementary file 1 — Supporting Information [file EM-56-684-s001.docx]

**Supporting information**

Table S1. Analysis of correlations (Spearman’s rho correlation) between study characteristics, 8-oxodG, telomere length and DNA methylation.

|  | Age | BMI | Respirable dust welders controls | Size of town for living | Ethnicity | Education | Cancer | Family history of cancer | Vegetable intake frequency | Fruit intake frequency | Fish intake frequency | Physical activity | Snuff | Smoking history | Passive smoking | Wine | Other alcohol | Wood burn at home | Wood Smoke neighbour-hood | Traffic intensity from house window | Time on traffic everyday | Hobby exposure welding fume | Hobby exposure engine exhaust | Hobby exposure dust |
| --- | --- | --- | --- | --- | --- | --- | --- | --- | --- | --- | --- | --- | --- | --- | --- | --- | --- | --- | --- | --- | --- | --- | --- | --- |
| 8-oxodG | 0.023 | -0.090 | 0.11 | 0.14a | 0.079 | 0.060 | -0.10 | -0.028 | -0.0072 | 0.025 | 0.041 | -0.029 | 0.048 | -0.056 | 0.075 | -0.011 | -0.026 | 0.081 | -0.083 | 0.062 | -0.0085 | -0.10 | -0.019 | 0.11 |
| Telomere length | -0.10 | -0.11 | -0.11 | -0.096 | -0.025 | 0.086 | -0.069 | 0.067 | 0.032 | 0.030 | 0.039 | 0.035 | -0.0071 | -0.0089 | 0.13a | -0.050 | 0.020 | -0.078 | -0.014 | -0.052 | 0.034 | -0.020 | -0.10 | 0.055 |
| Methylation *HOXA9* | 0.018 | -0.11 | 0.13a | 0.13a | 0.13a | -0.0077 | 0.020 | -0.049 | 0.092 | -0.0010 | 0.049 | 0.030 | 0.081 | 0.053 | -0.0015 | 0.045 | -0.035 | 0.11 | 0.072 | 0.0032 | -0.062 | 0.053 | -0.099 | 0.016 |
| Methylation *SHOX2* | 0.15a | 0.091 | -0.055 | -0.073 | -0.0060 | -0.033 | -0.081 | 0.032 | -0.040 | 0.022 | -0.056 | -0.13a | -0.088 | 0.017 | -0.012 | -0.057 | 0.034 | -0.10 | -0.051 | 0.065 | -0.065 | -0.12 | -0.15 | -0.068 |
| Methylation *CDKN2A* | 0.063 | 0.029 | 0.11 | 0.12 | -0.035 | -0.0049 | -0.068 | 0.035 | -0.037 | 0.017 | -0.059 | -0.052 | -0.013 | 0.054 | -0.055 | -0.091 | 0.020 | 0.028 | 0.078 | -0.0025 | 0.00050 | 0.098 | 0.051 | 0.015 |
| Methylation *MGMT* | 0.10 | 0.021 | -0.016 | -0.0032 | 0.081 | 0.028 | -0.040 | -0.0064 | -0.0030 | 0.072 | 0.086 | -0.0036 | -0.059 | -0.040 | -0.075 | -0.073 | -0.11 | -0.042 | -0.025 | -0.044 | -0.052 | -0.063 | 0.020 | 0.039 |
| Methylation *APC* | 0.014 | 0.055 | 0.16a | 0.14a | -0.038 | -0.14a | -0.019 | -0.072 | 0.098 | 0.10 | 0.017 | -0.085 | -0.029 | 0.081 | -0.035 | 0.12 | 0.018 | 0.19b | -0.018 | 0.0000 | -0.11 | -0.049 | 0.16 | -0.073 |

a P<0.05

b P<0.01

Table S2. Analysis of correlations (Spearman’s rho correlation) for oxidative stress, telomere length, and DNA methylation.

|  | 8-oxodG | Telomere | *HOXA9* | *SHOX2* | *CDKN2A* | *MGMT* | *APC* |
| --- | --- | --- | --- | --- | --- | --- | --- |
| 8-oxodG | 1.0 |  |  |  |  |  |  |
| Telomere length | 0.00069 | 1.0 |  |  |  |  |  |
| Methylation *HOXA9* | 0.11 | 0.013 | 1.0 |  |  |  |  |
| Methylation *SHOX2* | -0.014 | -0.11 | -0.17b | 1.0 |  |  |  |
| Methylation *CDKN2A* | 0.066 | 0.056 | 0.084 | 0.068 | 1.0 |  |  |
| Methylation *MGMT* | 0.069 | -0.10 | 0.0031 | -0.099 | -0.029 | 1.0 |  |
| Methylation *APC* | 0.079 | -0.11 | 0.044 | -0.16a | 0.042 | 0.0094 | 1.0 |

a P<0.05

b P<0.01
